# Massive A-to-I RNA editing is common across the Metazoa and correlates with dsRNA abundance

**DOI:** 10.1186/s13059-017-1315-y

**Published:** 2017-10-02

**Authors:** Hagit T. Porath, Binyamin A. Knisbacher, Eli Eisenberg, Erez Y. Levanon

**Affiliations:** 1 The Mina and Everard Goodman Faculty of Life Sciences, Bar-Ilan University, Ramat-Gan, Israel; 2 Raymond and Beverly Sackler School of Physics and Astronomy, and Sagol School of Neuroscience, Tel Aviv University, Tel Aviv, 69978 Israel

## Abstract

**Background:**

Adenosine to inosine (A-to-I) RNA editing is a post-transcriptional modification catalyzed by the ADAR (adenosine deaminase that acts on RNA) enzymes, which are ubiquitously expressed among metazoans. Technical requirements have limited systematic mapping of editing sites to a small number of organisms. Thus, the extent of editing across the metazoan lineage is largely unknown.

**Results:**

Here, we apply a computational procedure to search for RNA-sequencing reads containing clusters of editing sites in 21 diverse organisms. Clusters of editing sites are abundant in repetitive genomic regions that putatively form double-stranded RNA (dsRNA) structures and are rarely seen in coding regions. The method reveals a considerable variation in hyper-editing levels across species, which is partly explained by differences in the potential of sequences to form dsRNA structures and the variability of ADAR proteins. Several commonly used model animals exhibit low editing levels and editing levels in primates is not exceptionally high, as previously suggested.

**Conclusions:**

Editing by ADARs is highly prevalent across the Metazoa, mostly targeting dsRNA structures formed by genomic repeats. The degree to which the transcriptome of a given species undergoes hyper-editing is governed by the repertoire of repeats in the underlying genome. The strong association of RNA editing with the long dsRNA regions originating from non-coding repetitive elements is contrasted by the almost non-existing signal seen in coding regions. Hyper-edited regions are rarely expressed in a non-edited form. These results support the notion that the main role of ADAR is to suppress the cellular response to endogenous dsRNA structures.

**Electronic supplementary material:**

The online version of this article (doi:10.1186/s13059-017-1315-y) contains supplementary material, which is available to authorized users.

## Background

Adenosine-to-inosine (A-to-I) RNA editing is a fundamental post-transcriptional gene regulatory mechanism, diversifying the transcriptome of Metazoa [1, 2]. It is catalyzed by the family of adenosine deaminases acting on RNA (ADAR) enzymes [3] and is considered to be more active in the brain [4]. Editing in the coding region of a transcript can lead to an amino acid substitution (recoding), resulting in a novel protein isoform and, possibly, an altered protein function. Additionally, editing in the non-coding region of a transcript can affect splicing, microRNA targeting, RNA degradation, translation, and other important cellular processes [2]. Hence, the A-to-I editing pathway is tightly intertwined with other gene regulatory networks operating in the cell. Inactivation or deletion of ADARs in various model organisms result in lethality or severe phenotypes, including aberrant embryonic development, pleiotropic defects, and neurological and behavioral phenotypes [5–8]. In human, alterations in editing levels were linked to various diseases, including cancer [9–11]. Collectively, these studies emphasize the importance of ADAR-mediated RNA editing to development, aging, and tissue homeostasis.

Advancements in sequencing technology accompanied by development of algorithmic methods enabled systematic studies of RNA editing, thereby revealing the vast scope of the editing in both vertebrates and invertebrates. The number of editing sites varies considerably across species. Over 5000 editing sites have been uncovered in *Drosophila* [12–15], while almost 50,000 sites have been found in *C. elegans* [16]. For mammals, 40,000 A-to-I editing sites have been reported in mouse [17, 18] and millions in human [19–26]. Strikingly, the vast majority of editing sites found to date occur in non-coding regions of the genome. For example, only a few thousand sites in human coding sequences were found so far, most of them weakly edited, and only a few dozen sites are conserved across mammals [27]. Virtually all editing activity is located in non-coding repetitive elements, which readily pair with inverted copies of the same repeat to form double-stranded RNA (dsRNA) substrates that are the preferred targets of the editing enzymes [28]. Even in cephalopods, for which tens of thousands of recoding sites were observed [29, 30], the vast majority of messenger RNA (mRNA) editing activity occurs in non-coding regions.

For a long time, it was believed that the main functional impact of RNA editing is its recoding capacity, resulting in the introduction of novel proteins. This was corroborated by the rescue of the ADAR2 knockout phenotype in mouse by inserting into the genome the edited version of a single recoding site in the *gria2* gene [6]. However, recent evidence indicates that a critical role of ADAR1 during mammalian development, and possibly an essential function of editing along metazoan evolution, is editing of non-coding dsRNAs [31, 32]. Specifically, embryonic lethality of ADAR1 knockout in the mouse can be rescued by concurrent deletion of MDA5, a receptor that recognizes long dsRNAs as non-self and triggers an innate immune response as part of the organism’s antiviral defense mechanism [33–35].

Editing by ADARs is found across Metazoa, starting with the earliest-diverging eumetazoan phyla [36], corals. However, not much is known about the scope of editing activity and its evolution across species. Transcriptome-wide screens for editing have been conducted for a number of species, but they are severely limited by technical requirements (see below). Furthermore, comparing the editing level between different species is complicated by non-trivial normalization issues (variations in coverage and read length, different source tissues, quality and length of the underlying genome reference sequence, availability of comprehensive Single-nucleotide polymorphism (SNP) mapping, etc.). Several inter-species comparative analyses have been published, though. It was shown that primate genomic repeats are being edited to a large extent, far more than mouse and fly [37], possibly due to properties of the Alu elements [38]. Recently it was shown that cephalopods are exceptional in the amount of editing in their coding sequence [29]. However, not much is known about the general scope of editing across the metazoan lineage, how frequent editing is in the typical organism, and what controls the level of ADAR activity.

**Fig. 1:**
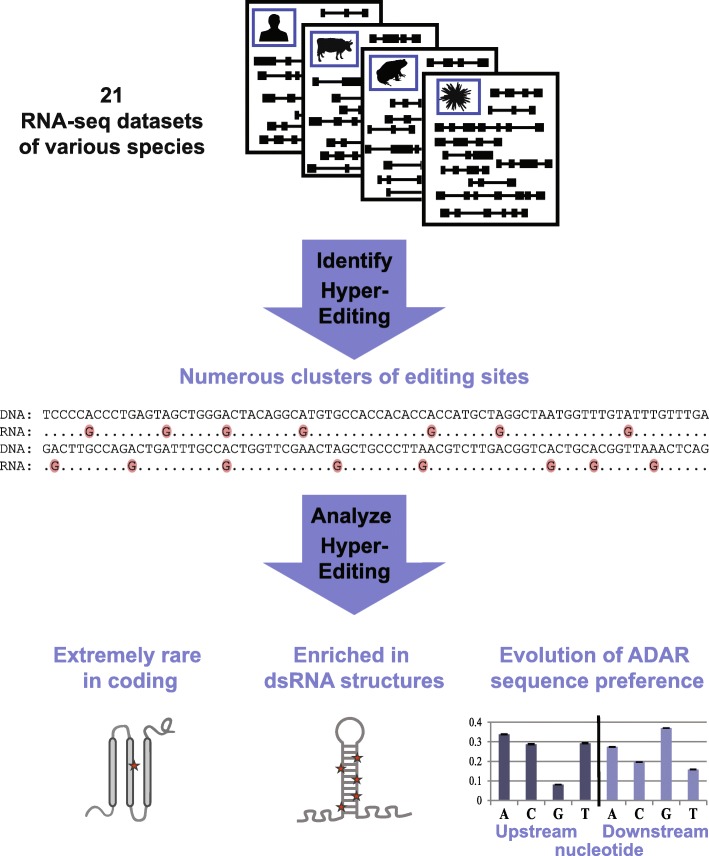
Overview: analyzing hyper-editing across species. RNA-seq datasets of 21 species were screened for clusters of RNA A-to-I editing, using the hyper-editing pipeline [39]. The identified hyper-editing sites were then characterized, revealing an enrichment in putative dsRNA structures and evolution of the ADAR sequence preference

Here we employ a recently developed approach for detection of hyper-edited reads [39] to compare the transcriptome-wide level of editing in clusters of sites in brain tissues (where applicable) originating from 21 eukaryotic species, from yeast to mammals (Fig. 1). The method does not pose any specific requirements on the underlying data except for having an RNA-sequencing (RNA-seq) dataset and a corresponding genome reference sequence and allows for a simple normalization and convenient comparison across species. Using this method, we find numerous sites for multiple species and show that the level of editing in clusters is determined, by and large, by the properties of the genomic repetitive elements.

## Results

### Numerous hyper-editing sites in various species

Standard approaches for editing detection depend on the availability of matching DNA and RNA samples from the same individual animal (or, alternatively, multiple RNA-seq samples with high coverage [15]), a curated genome reference sequence, as well as a database of common SNPs. The main obstacle for a comprehensive survey of editing in multiple species is the availability of these data, typically obtainable only for a few model organisms.

A recently published algorithm for detecting hyper-edited reads [39, 40] provides a highly specific method to detect RNA editing, independently of DNA-seq data or any prior knowledge about SNP data, and applicable to any coverage level. Hyper-edited reads harbor large clusters of editing sites. Aligning these reads to the genome results in clusters of DNA–RNA mismatches that are distinctive compared with sequencing errors, SNPs, and other sources for DNA–RNA mismatches. However, standard alignment tools often fail to align these reads properly, due to the large number of mismatches, and they are usually discarded. The hyper-editing computational screen focuses on the reads that fail to align by standard tools and realigns them after pre-masking potential editing sites [39].

**Fig. 2:**
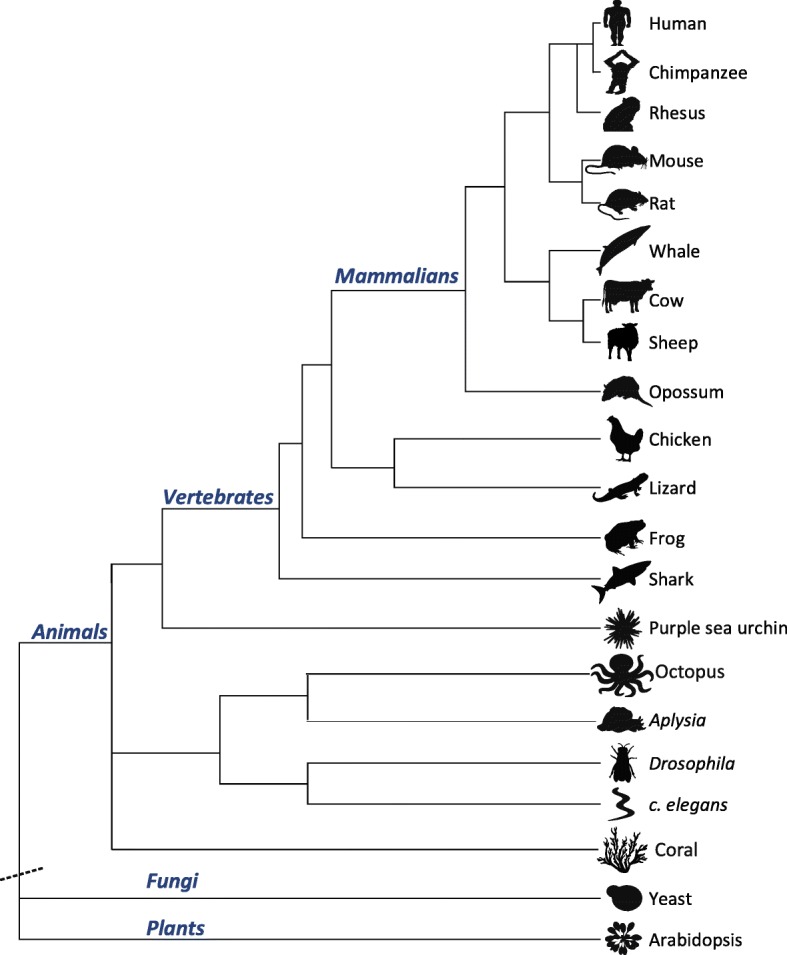
Phylogenetic tree of the studied organisms (based on the UCSC Genome Browser [59]). The lengths of branches in the phylogenetic tree are not drawn to scale

Here we employ this approach to quantify the level of hyper-editing in 19 different animal species, ranging from coral to human (Table 1 and Fig. 2), focusing on the highly edited brain tissues (when applicable). As detailed below, numerous editing sites are observed in all these metazoan species that contain the *adar* gene. As a control, we have also looked at *Saccharomyces cerevisiae* and *Arabidopsis thaliana* that do not contain the ADAR editing enzymes and verified that they do not show any evidence for A-to-I hyper-editing (see Table 1). The following results refer to the 19 metazoan species, excluding yeast and *Arabidopsis*.

**Table 1:** Editing values across 21 species

Organism^a^	Tissue/Source	Source reads^b^ [% aligned]	Hyper-editing reads	Editing events	Unique editing sites [% A-to-G of all types]	Unique sites overlapping with coding regions^c^ [% of total]	Unique sites overlapping with repeats^c^ [% of total]
Human	Brain	64,313,204 [92]	22,117	117,383	75,984 [99]	31 [0]	70,160 [92]
Chimpanzee	Brain	20,083,064 [66]	2327	10,866	9916 [95]	76 [1]	9089 [92]
Rhesus	Brain	215,339,102 [87]	169,735	921,561	525,245 [91]	1796 [0]	499,388 [95]
Mouse	Brain	114,374,684 [90]	5784	28,910	13,748 [94]	99 [1]	9497 [69]
Rat	Brain	238,077,800 [84]	23,724	119,260	31,788 [87]	1096 [3]	23,562 [74]
Minke whale	Brain	51,470,260 [94]	20,301	180,079	121,897 [100]	701 [1]	106,429 [87]
Cow	Brain	208,706,410 [87]	70,520	389,830	200,770 [99]	1161 [1]	188,187 [94]
Sheep	Brain	31,846,364 [91]	15,792	83,756	17,316 [93]	84 [0]	15,198 [88]
Opossum	Brain	69,848,223 [65]	613	2926	2168 [90]	132 [6]	1786 [82]
Chicken	Brain	269,226,888 [88]	24,690	169,793	79,728 [98]	2991 [4]	42,718 [54]
Lizard	Brain	183,282,934 [60]	98,483	700,905	122,793 [98]	284 [0]	52,343 [43]
Frog	Brain	51,896,478 [75]	54,519	388,744	147,172 [97]	180 [0]	69,943 [48]
Elephant shark	Brain	139,569,606 [75]	67,704	449,965	200,171 [89]^d^	428 [0]	162,066 [81]
Purple sea urchin	Young juvenile	76,613,634 [71]	36,570	216,215	83,594 [80]^d^	239 [0]	25,661 [31]
Octopus	CNS	344,308,354 [86]	1,135,890	7,851,521	1,053,826 [99]	-	-
Sea hare (*Aplysia*)	CNS	63,075,904 [77]	2952	23,757	12,546 [95]	2 [0]	936 [7]
Fly (*Drosophila*)	Head	257,255,489 [97]	6124	57,065	39,472 [100]	1124 [3]	14,471 [37]
Nematode (*C. elegans*)	Larvae	133,158,570 [97]	8691	65,543	21,713 [100]	244 [1]	11,782 [54]
Coral	WT	65,782,768 [61]	47,546	314,345	127,069 [88]	-	-
Yeast (*S. cerevisiae*)	WT	23,339,332 [99]	7	42	12 [63]	-	-
Thale cress (*Arabidopsis*)	WT	49,166,984 [96]	5	26	11 [13]	-	-

^a^Additional details are given in the table in Additional file 2

^b^All samples were run as single-ended and strand-indifferent (for comparison reason)

^c^We annotated coding regions using xenoRefGene (RefSeq for Human and Frog) and repeats regions using RepeatMasker, both from the UCSC Genome Browser. Octopus and Coral were omitted from this analysis since their genomes and annotations are not available in the UCSC Genome Browser

^d^A-to-C signal with sequencing error features was also identified in the sample; thus, for specificity calculations A-to-C sites were excluded

Altogether, we analyzed ≈ 2.5 × 10^9^ RNA-seq reads (range of 72–101 bp of length). Of these, the Burrows-Wheeler Aligner (BWA) could not find any alignment to the reference genome for ≈ 4.2 × 10^8^ reads (17%). Most of the unmapped reads originate from poorly annotated or highly variable genomic regions, identically duplicated genomic regions, contamination of bacterial and viral RNAs, or reflect various technical issues (mainly sequencing errors) [41]. However, we found that about 0.4% of these are unmapped due to extensive RNA editing that changes the RNA to a point it is not recognized by standard alignment tools as originating from the DNA sequence. Using our algorithm, we could map 1,849,214 such hyper-edited reads, containing 12,222,117 editing events (2,832,779 unique genomic sites).

**Fig. 3:**
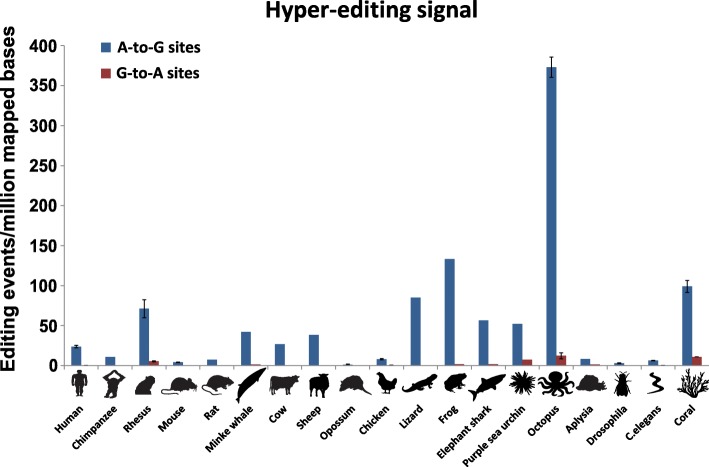
Comparing the normalized hyper-editing signal for 19 different species. The level of hyper-editing is measured by the number of A-to-G mismatches in the identified clusters per million mapped bases (standard errors bars are presented for nine species for which we had biological replicates, Additional file 1: Figure S8). For comparison, the numbers of G-to-A clusters (found using the same parameters) are presented, representing the expected false-positive rate

The specificity of the detection screen is evaluated by comparing the number of clusters of mismatches that are presumably due to A-to-I editing with the abundance of clusters of other types of mismatches. We find that 80–100% of the unique cluster sites (94% average per organism) belong to A-to-G mismatch clusters (see Table 1 and Fig. 3). Note that some of the datasets are not stranded (i.e. one cannot tell which strand of the cDNA corresponds to the expressed RNA), and thus the hyper-edited clusters may appear as T-to-C mismatches. In these cases, similar numbers of A-to-G and T-to-C clusters are observed. In contrast, for the stranded samples virtually only A-to-G clusters are seen, as expected (Additional file 1: Figure S1). The number of clusters of mismatches other than A-to-G is comparable to the numbers observed in the control species lacking ADAR enzymes (yeast and *Arabidopsis*) (see Table 1).

In order to compare editing levels across species, we looked at the number of hyper-editing events observed per million mapped bases as an approximated normalized measure of the true hyper-editing rate (normalized hyper-editing signal). Most of the available datasets consisted of 70–80-bp-long reads. To minimize the technical variability, the comparative study of hyper-editing levels was done for reads of this length. We included datasets with longer reads, but trimmed their starts prior to the hyper-editing cluster search, to allow for an unbiased comparison.

The normalized hyper-editing signal was found to vary considerably between the different species studied (Fig. 3). The number of hyper-edited reads was in the range of 613–1,135,890 reads per organism (median 23,724), and these reads contained 2926–7,851,521 editing events per organism (median 180,079), residing at 2168–1,053,826 unique genomic sites per organism (median 75,984) (see Table 1 and Additional file 2). Similar results were observed in other tissues (Additional file 1: Figure S2).

### Evolution of the ADAR recognition motif

**Fig. 4:**
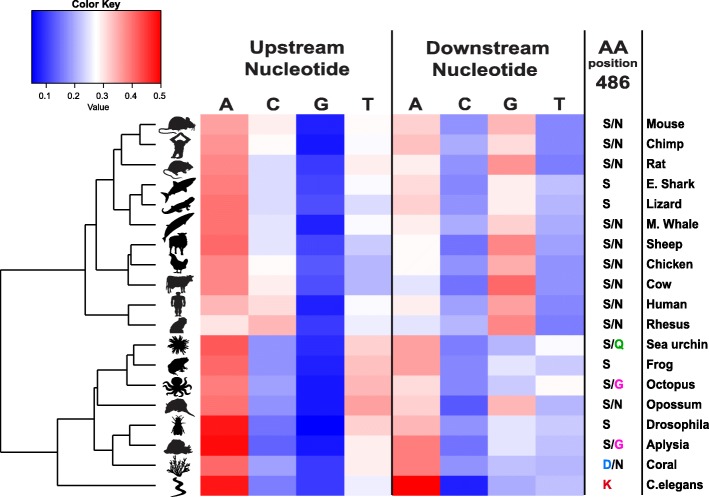
Evolution of the ADAR sequence preference, based on the sequence context (upstream and downstream adjacent locations) of the hyper-editing sites. The motifs cluster into two groups, largely consistent with their phylogeny: most vertebrates cluster together whereas amphibian and invertebrates cluster to a different group (with one exception, opossum, which has a small number of sites and possibly a noisy motif). In both clusters, G is depleted in the upstream nucleotide, whereas the downstream nucleotide preference is different for the two clusters. The first cluster exhibits a preference for G downstream, whereas in the other one A is preferred. (*Red*: over-representation; *blue*: under-representation). The downstream nucleotide preference is determined by the residue S486 (PDB structure 5HP2), as described by Matthews et al. [45]. Presented here for each organism are the amino acids observed at this position (see also Additional file 1: Figure S3). The five species exhibiting the most different 3' preference encode ADARs with a different amino acid in this position

Analysis of the sequence context surrounding our detected sites across species reveals an evolution of the ADAR sequence preference (Fig. 4). Looking at the two-neighbor preferences (one base upstream of the site and one base downstream) per species, we found that the species cluster into two distinct groups, largely consistent with their phylogeny: mammalians and reptiles are clustered together, whereas the amphibians and invertebrates are clustered to a different group, with a single exception (the marsupial opossum, with a low number of editing sites resulting in poor motif statistics). Both clusters share the strong depletion of G upstream, in agreement with the known ADAR sequence preference for the few organisms studied so far [42, 43], but have different preferences at the downstream nucleotide. ADARs of the first group (mammals and reptiles) prefer G downstream, while the second group of organisms exhibits enrichment of A in that position. This is consistent with the observed motifs of *C. elegans* [44] and the *A. millepora* coral [36]. Although the motif reported here is based only on hyper-edited sites, it is largely consistent with the known ADAR recognition motif. However, we cannot rule out the possibility of some subtle differences in sequence preferences between the hyper-edited sites and other editing sites. Analyzing this question would require a large-scale mapping of all editing level across species.

A recently published ADAR protein structure enables us to correlate this observed variation in the motif across Metazoa with sequence variations. The crystal structure of human ADAR2’s deaminase domain bound to dsRNA (5HP2), reported by Matthews et al. [45], shows that the downstream G preference is a result of a direct interaction between the G nucleotide and Serine 486 (S486). Multiple sequence alignment of ADAR proteins across Metazoa (Additional file 1: Figure S3) reveals that this S486 is conserved in ADAR2 across species and is substituted with Asparagine, a different polar residue, in ADAR1. Intriguingly, the only five species whose genomes encode ADARs with different amino acids at this position are those with the greatest reduction in downstream G preference (20–25% of edited sites vs. 35% in G-preferring group): urchin encodes glutamine, *C. elegans* encodes lysine, octopus and aplysia encode glycine, and coral encodes aspartic acid. Of these, *C. elegans* has the weakest preference for G downstream (20% of sites), possibly because it is the only one of these species that does not encode an additional ADAR harboring a canonical serine/asparagine.

### Hyper-editing is extremely rare in coding sequences and is abundant in repeats

Although hyper-editing is common, it is rarely found in coding regions for all tested species (Table 1), consistent with previous findings in several vertebrates [39]. Less than 1% of the sites were found in coding regions for most of the organisms studied, with the exceptions of *Drosophila* (3%), rat (3%), chicken (4%), and opossum (6%). Although coding annotations for most of the species are imperfect, these results strongly suggest that for all species, RNA hyper-editing by ADARs primarily occurs in the non-coding part of the transcriptome.

The vast majority of editing events in primates and mouse is known to reside in repetitive element [46–51]. These regions are more likely to hybridize with nearby oppositely oriented repeats, creating the dsRNA structures required for ADARs binding. Here, too, we find that 77% of all detected hyper-edited sites reside within annotated repeats of the respective species (Table 1, Additional file 1: Figure S4). For most organisms studied, the majority of hyper-edited sites are localized in repetitive sequences derived from mobile elements. Note that the accuracy of repeats annotations varies across species (remarkably inferior for the less researched animals), which could account for the relatively low percentage observed in some species (e.g. sea hare and purple sea urchin). A notable exception is the fly: although its genome and repetitive elements are well annotated, only 37% of its hyper-editing sites are found in its repeat regions, in agreement with a previous report [14]. This result is consistent with the fly having only a single ADAR enzyme, an ADAR2 ortholog, which is considered to be responsible for the majority of editing of mammalian coding sites, and is known to be capable of editing short and imperfect dsRNA structures.

SINE is known to be the most edited repeat class in primates [25]. SINE repeats are highly widespread in genomes and tend to be similar to each other. In contrast, typical copies of mammalian LINEs are fragments originating from random parts of the full-length consensus LINE sequence. For example, two neighboring human L1 LINE fragments typically correspond to different parts of the consensus and cannot form dsRNA structure. Indeed, we find that most of the edited clusters reside in SINEs. An interesting exception to this rule is the CR1 LINE in chicken with over 35 K unique sites in this repeat, over fivefold more than all other repeats combine. Although the full length of *CR1* is ~4.5 kb, only a few dozen copies of the repeat are full length and the vast majority of the repeats consist of a small part of the 3’ of the full LINE [52] (average length 334 bps). Thus, two typical CR1 copies are both derived from the 3’ end of the consensus and are therefore likely to form dsRNA, effectively behaving like SINEs (in this aspect). The only additional massively edited LINE is the Penelope element in lizard with over 40 K unique sites (compared with 8663 sites in all SINEs).

### ADAR tendency to dsRNA structure

In order to further support the association of the loci detected as being hyper-edited to dsRNA structures binding ADARs, we tested whether the detected sites' loci do indeed form dsRNA. We used pairwise BLAST alignment (*bl2seq* [53]) to look for putative long and strong dsRNAs (≥65% identity along ≥ 80% of the hyper-edited cluster) formed by the hyper-edited loci and their flanking genomic sequence (±2 kb) (see “Methods”), focusing on five representative species (human, chimpanzee, cow, lizard, and frog). As expected, we could detect putative long and strong structures surrounding a large fraction of the detected hyper-editing clusters: 49.6% ± 9.5 (mean over the five species ± std) of the hyper-edited loci reside within these putative structures, compared with only 21.1% ± 12.1 (mean ± std) for the control search (see “Methods”).

Two species have an exceptionally high rate of hyper-editing (Fig. 3), *Octopus bimaculoides*, known to have an overall elevated editing activity [29], and the frog *Xenopus tropicalis*. In order to test whether the elevated hyper-editing signal can be attributed to abundance of dsRNA structures, we measured the probability of a random 50-bp-long genomic sequence to form a long stable dsRNA structure with its surrounding genomic sequence (see “Methods”) for all 19 species. Indeed, the fraction of loci putatively creating long, nearly perfect dsRNA in octopus and frog (4.4⋅10^–3^ and 3.2⋅10^–3^, respectively) is exceptionally high compared with almost all other species (Additional file 1: Figure S5). It should be stressed that the above described measurement is not expected to be a faithful measure of the abundance of dsRNA, as we do not take into account the widely distributed expression levels of the genomic regions (reliable transcriptomes and expression profiles are not available for most species studied). Furthermore, the detailed properties of the dsRNA structures (length, tightness of structure) as well as ADARs’ efficacy and expression also vary across species. Thus, one should not expect a linear dependence between the fraction of loci putatively creating dsRNAs and the measured hyper-editing signal. Nevertheless, the correlation between the two is a strong indication towards the role played by long, nearly perfect dsRNAs in hyper-editing.

**Fig. 5:**
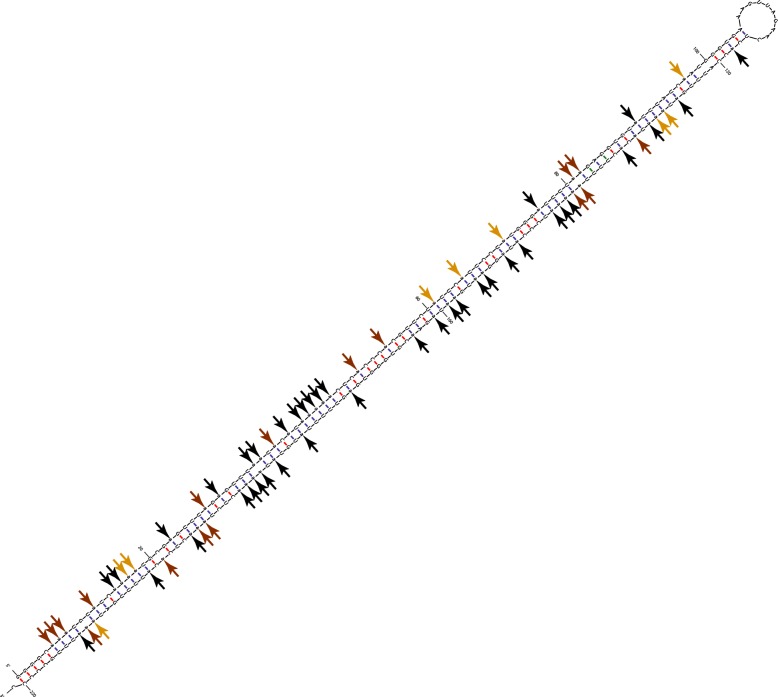
*Harbinger* is the most edited repeat family in *Xenopus tropicalis*, belonging to the DNA repeat class. The *Harbinger* repeats are palindromic, likely forming tight dsRNA structures. Here we show the predicted secondary structure (using MFOLD [60]) for a single representative *Harbinger* repeat (221-bp in length; located at GL172703: 562862-563082) which was found to be highly hyper-edited (65/77 adenosines were found hyper-edited; marked with arrows). Clearly, tight dsRNA is formed without the requirement of nearby reverse-oriented similar repeat, explaining the high level of hyper-editing in *Xenopus tropicalis*. We measured the editing level for each site (using all reads, including ones that were normally aligned to the region). Strongly edited sites (>30%) are marked with red arrows, moderately edited sites (1–30%) with orange arrows, and black arrows point to sites that were not found edited by the non-hyper-edited reads (or were not covered by those reads), see also Additional file 1: Figure S7

The abundance of dsRNAs in the frog can be partially explained by the observation that its most edited repeat family, the ~200-bp *Harbinger* (a DNA repeat class), has a palindromic consensus sequence. Thus, these repeats can fold to create a very tight dsRNA structure [54] (Fig. 5) and do not require the existence of a nearby, similar, reverse-oriented repeat to act as a favorable ADAR target. Indeed, we verified that 30% of the edited Harbinger repeats do not have any neighboring inversely oriented repeat within 5 kb. As the Harbinger dsRNAs are formed by folding of the repeat on itself (see Additional file 1: Figure S6), the loop is rather short, leading to an elevated editing level [38], resulting in extensive editing of these repeats and their surrounding regions [55].

Thus, our results show that the majority of hyper-editing activity is associated with long, nearly perfect dsRNA structures. Most of these structures are formed by pairing of neighboring inverted repetitive elements. This scenario was shown in detail for several model organisms, and our present results support its general relevance to all Metazoa.

### Hyper-edited regions typically do not express unedited transcripts

Interestingly, the vast majority of hyper-edited loci in all genomes studied (60–70% of the sites, Additional file 1: Figure S7) support no other reads but the hyper-edited ones. That is, we observe no additional reads aligned to the same locus that do not harbor a large number of editing sites. In other words, these loci are rather weakly expressed and the transcripts that are expressed from them are virtually all extensively edited. Each hyper-edited read contains at least four edited sites (the average is higher, 6.62 sites per hyper-edited read) and one should expect a similar number of edited sites on the other strand of the edited dsRNA structure, doubling the number of sites in each edited substrate. As the typical length of reads studied here is 80 bp, we conclude that at least 10% (16% on average) of the base pairs in these hyper-edited dsRNA regions are edited. Thus, hyper-editing should be very effective in unwinding the long, nearly perfect dsRNAs at which it occurs.

Taken together with the results of the previous section, these observations are consistent with the recent view that a primary function of the ADAR enzymes is to destabilize long, nearly perfect self dsRNA through extensive A-to-I editing, thus preventing false stimulation of the innate immune system and triggering the interferon cascade [33–35, 56].

## Discussion

ADAR enzymes are expressed in all Metazoa studied so far. Here we quantified their editing activity in a wide panel of animals, looking at clusters of RNA editing sites. We found that editing is observed in all species expressing ADARs, but its abundance varies considerably. Furthermore, the scope of hyper-editing is determined, by and large, by the genomic potential for creating long, nearly perfect dsRNAs. Accordingly, the clusters of editing sites reside mainly in repetitive elements, which are the main source for such dsRNA structures. When two similar inverted copies of the same repeat reside in the same pre-mRNA molecule they may pair together and be edited by ADARs. Alternatively, when the repetitive element itself is approximately a palindrome, as is the case in the Harbinger repeat of the frog, it may form dsRNA and be edited even in the absence of a nearby inverted repeat. The ADAR enzymes then unwind nearly all copies of these long, nearly perfect, double-stranded transcripts (for the majority of the detected regions) by extensive editing (Additional file 1: Figure S7).

Millions of sites were identified in the human genome, more than in any other species studied so far. However, using our quantitative approach for inter-species comparison, we find that human is not exceptional in terms of its hyper-editing activity. Interestingly, a while ago we have shown [37] (based on a very small dataset available) that human shows many more clusters of editing sites compared with mouse, rat, chicken, and fly, and suggested human (or primates) may be unique in its editing behavior. While the results for these five species still hold, the present comparison against a broader spectrum of species makes it clear that hyper-editing in human does not stand out in any way. Remarkably, the common model animals tested here (mouse, rat, fly, and *C. elegans*) have much lower editing levels than other animals studied (for the latter two, a partial explanation may be the fact that the coding fraction of their transcripts is high compared with other animals studied, leaving less room for repetitive elements). Thus, the scope of editing in a typical species is higher than was assumed so far based on the model organisms studied. The two organisms showing the strongest hyper-editing signal are octopus, which is known to have a unique editing behavior in general, and the frog that harbors a palindromic, heavily edited, repeat.

The discrepancy between the large number of documented sites and the relatively modest ranking of human in terms of the hyperediting signal may suggest that the number of reported human sites, larger than for any other species, reflects nothing but the availability of much more expression data compared to other species. Alternatively, it is possible that quantification of hyper-editing, clusters of sites in the same RNA molecule, is not always a good proxy for the global editing activity. Clearly, editing of isolated sites in the coding sequence is very much different than hyper-editing. In human, we have shown recently that the overall hyper-editing level is highly correlated with global editing activity [11], but this correlation might not hold for an inter-species analysis. Even the global editing level in repeats, mostly determined by molecules that are edited in one or few locations, could possibly show a different species-dependence than implicated by the number of multiply edited molecules. This point should be revisited in the future, as the required large-scale matched DNA and RNA data become available.

The activity of retroelements and other mobile genomic elements is an important driving force of genome evolution [57, 58]. As a result of this activity, repetitive elements accumulate in the genome, leading to increasing numbers of putative dsRNAs. As was recently demonstrated, these dsRNAs may trigger an undesired innate immune response and a primary role of the ADAR enzyme is to edit these structures in order to prevent this response [33–35]. Our results confirm that hyper-editing, concentrated in repetitive elements, has the potential to destabilize and eliminate the dangerous dsRNA structures. Therefore, this editing activity allows retroelements to be tolerated in the genome and thus plays a critical role in enabling this major driving force for genome evolution.

## Conclusion

Extensive A-to-I hyper-editing is a common feature in metazoans. The prime targets of ADARs are dsRNA structures formed by repetitive elements. The amount of such targets in the transcriptome varies considerably across species, and depends on the characteristics of the genomic repeats in the underlying genome. Interestingly, most of the hyperedited regions are rarely expressed in a non-edited form, suggesting that at least some of these may be the critical ADAR1 targets, whose editing is essential to suppress an undesired activation of the innate immune system by endogenous dsRNA structures.

## Methods

### Identification of hyper-editing reads and sites

Hyper-editing sites were identified as described previously [39], with default parameters. Briefly, the hyper-editing pipeline allows for picking up the contribution of heavily edited reads that differ so widely from the corresponding DNA to the extent that standard schemes fail to align them properly [39]. To identify such extensively edited reads, we apply the following simple but effective four-step approach: (1) collect all unmapped reads from the initial alignment; (2) transform all As to Gs in both the unmapped reads and the reference genome; (3) realign the transformed RNA reads and the transformed reference genome; and (4) recover the original sequences and search for dense clusters of A-to-G mismatches. The RNA-seq data we used are mostly strand-indifferent and, therefore, even for true A-to-G sites, the observed mismatches are either A-to-G or T-to-C with roughly equal amounts (Additional file 1: Figure S1). The specificity of an editing detection screen is usually gauged by rerunning the same pipeline, looking for dense clusters of identical mismatches of types other than A-to-G. Since non-A-to-G editing is expected to be extremely rare, the fraction of non-A-to-G clusters to all clusters provides a useful measure of the screen’s specificity (Fig. 3). Using our standard parameters, we call a read hyper-edited if the number of A-to-G mismatches exceeds 5% of its length (four sites for the 80-bp reads). Hyper-edited sites showed the familiar ADAR sequence preference, tend not to overlap known SNPs (unlike detected sites of non-A-to-G type), are uniformly distributed across read positions, and (when occurring within RefSeq transcripts) conform to the expressed strand.

As an input, we used 62 RNA-seq datasets for the various species, GEO/SRA IDs, and other details about the datasets are given in Additional file 2. The reference genomes were downloaded from the UCSC Genome Browser [59], assembly versions are specified in Additional file 2. We consider paired-end datasets as two separated single-end datasets and strand-specific libraries as non-stranded samples to make all datasets comparable.

### Genome, repeats, and coding regions annotations

Genome reference sequences were downloaded from UCSC and from NCBI (assembly information is given in Additional file 2). Coding regions were annotated using xenoRefGene tables (RefSeq for Human and *Xenopus tropicalis*) and known repeats were annotated using the RepeatMasker tables, all downloaded from the UCSC Genome Browser [59] in August 2014. The octopus and coral information is not available in UCSC and therefore the two species were excluded from the analyses that are based on these UCSC annotations.

### Defining hyper-edited clusters and dsRNA structure

Clusters of hyper-edited reads are defined as the part of the edited read starting at the first A-to-G mismatch and ending at the last one.

To detect potential dsRNA structure formed by hyper-edited RNAs, the DNA sequences matching the hyper-edited clusters were aligned to the genomic sequences 2 kbp upstream and 2 kbp downstream of the clusters. We used *bl2seq* [53] with parameters -F F -W 7 -r 2, to look for a reversely oriented sequence that is similar (at least 65% identity along 80% of the hyper-edited cluster length) to the hyper-edited cluster location. As a control, we looked within the same region (2 kbp upstream and downstream) for similar sequences (same parameters) that are present on the same strand (thus not forming dsRNAs).

To quantify the genomic potential to create dsRNA structures, we randomly chose 10,000,000 regions, each 50 bp long, and looked for highly similar (>95% identity at least 40 bp long), reverse-oriented sequences in the flanking genomic region (2 kbp upstream and 2 kbp downstream). Here, too, *bl2seq* was used, with the same parameters. As expected, the predicted dsRNA regions were highly enriched with editing events (average fold-change of 6.4) in all but one of the species.

## Additional files

Additional file 1: Supplementary Figures 1–8, Supplementary Table 2. (PDF 760 kb)

Additional file 2: Supplementary Table1. (XLSX 15 kb)
