# Supplementary material for: Massive A-to-I RNA editing is common across the Metazoa and correlates with dsRNA abundance
Source: Genome Biol. 2017 Oct 2;18:185. doi: 10.1186/s13059-017-1315-y (PMC5625713; doi:10.1186/s13059-017-1315-y)
Supplement: Supplementary file 1 — Supplementary Figures 1–8, Supplementary Table 2. (PDF 760 kb) [file 13059_2017_1315_MOESM1_ESM.pdf]

Strand distribution of hyper-edited sites

Cow: stranded sense reads | Cow: stranded antisense reads | Chimpanzee: Non-stranded reads

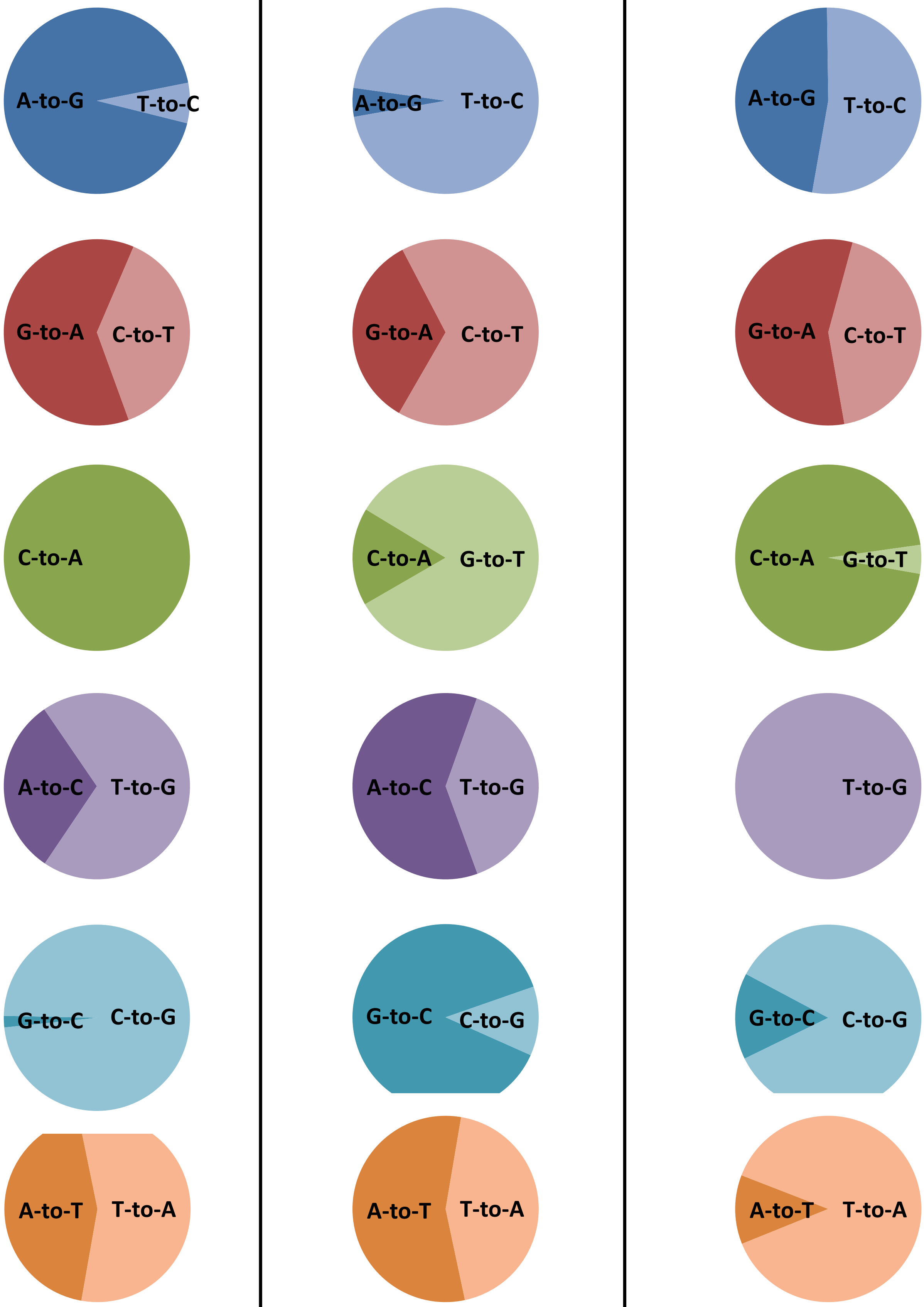

Supplementary Figure 1. **Strand distribution of the detected editing sites.** Pie charts showing the distribution of editing sites for the specified mismatch. Results of three different datasets are presented. The cow dataset was stranded and contains sense reads (sequence of the expressed strand) and antisense reads (sequence of the antisense of the expressed strand), whereas in the chimpanzee dataset the sequenced strand was arbitrary. Only the A-to-G sites show the expected behavior from genuine editing sites, namely, near 100% A-to-G preference at cow-sense reads (at left), near 0% at cow-antisense reads (at middle) and random distribution (≈50/50%) at the non-stranded chimpanzee sample (at right).

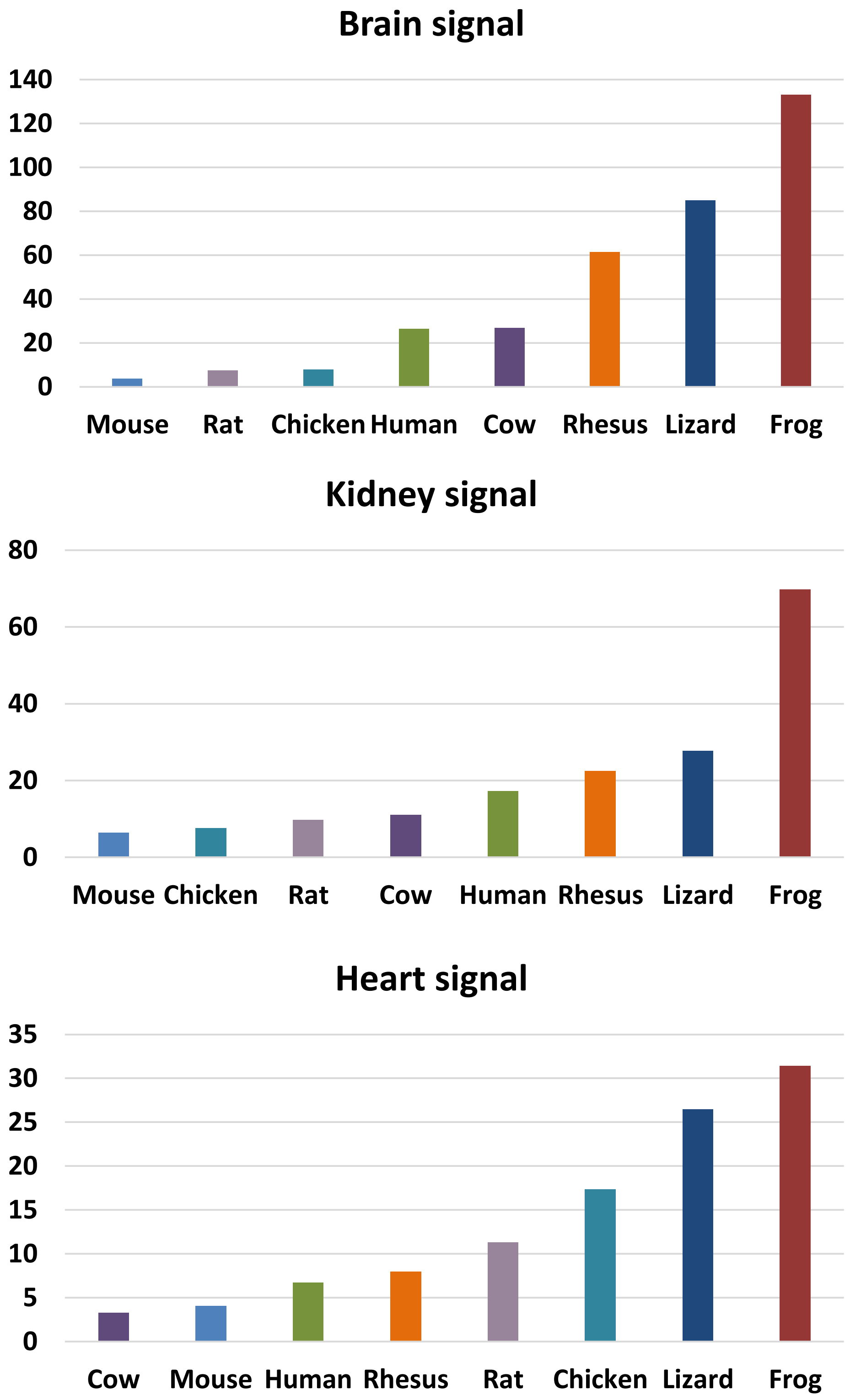

Supplementary Figure 2. Comparative study of Hyper-editing for three tissues. Normalized hyper-editing signals were calculated for kidney and heart samples of 8 species, and compared to the brain results. The absolute number of events per read varies considerably between brain and kidney, but the relative ranking of the species studied was largely maintained. The heart data shows a larger deviation of the relative ranking, which might be related to the overall low editing rate in this tissue.

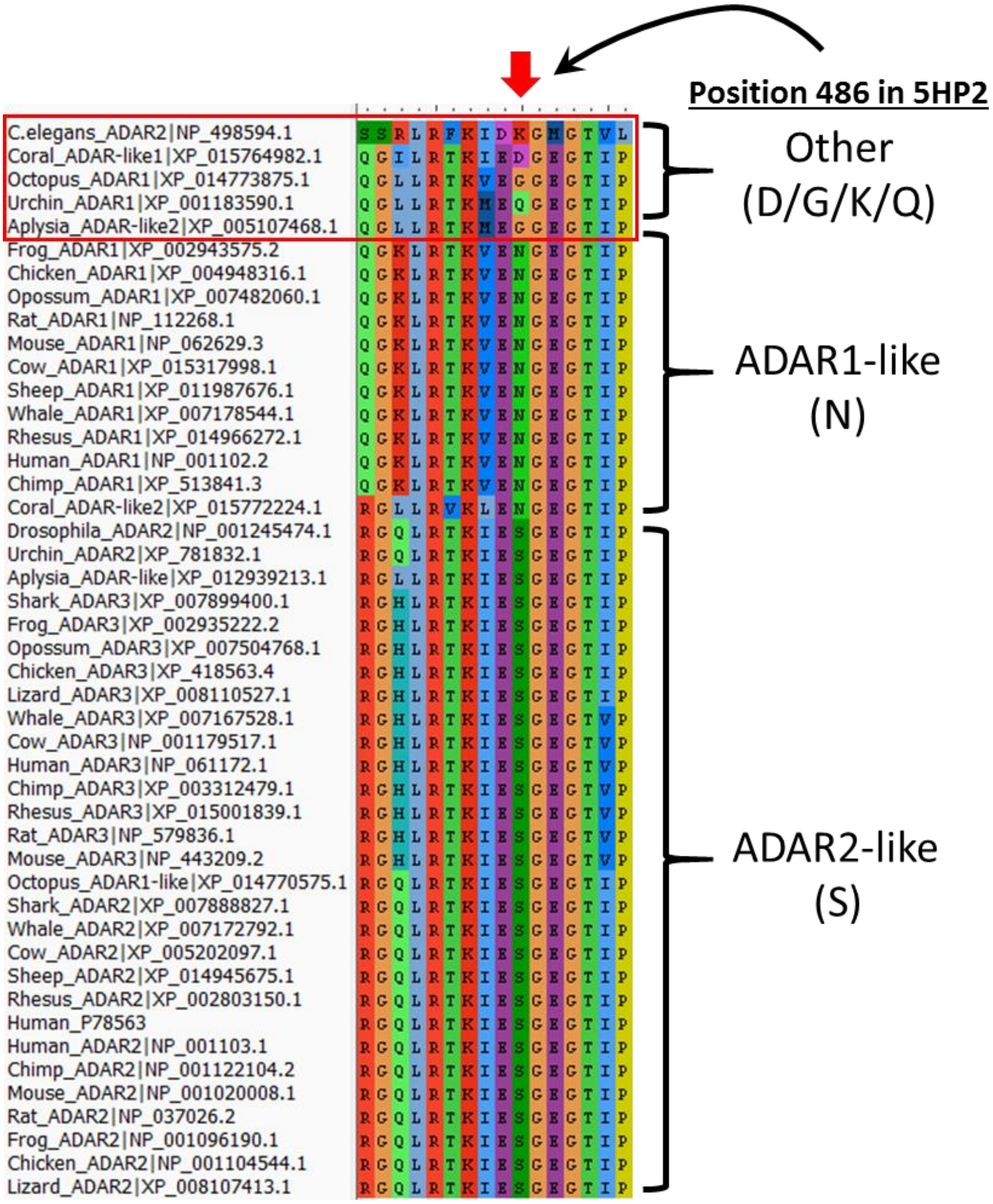

Supplementary Fig. 3. Multiple sequence alignment of ADAR proteins across species. Multiple sequence alignment (MSA) of the region responsible for the canonical downstream G preference of ADARs is presented. In particular, this preference is associated with the interaction between the downstream G and S486 in PDB structure 5HP2 (red arrow in MSA) described by Matthews et al. (Nat. Struct. Mol. Biol 2016). This position contains Serine in ADAR2/ADAR3 and Asparagine in ADAR1. However, the 5 species exhibiting a different 3' preference (see Figure 4), encoding ADARs that present a different amino acid in this position (red box). Sequences used for the alignment and respective annotation as ADAR1/2/3 were taken from NCBI's Protein database (see accession numbers in Supplementary Table 2).

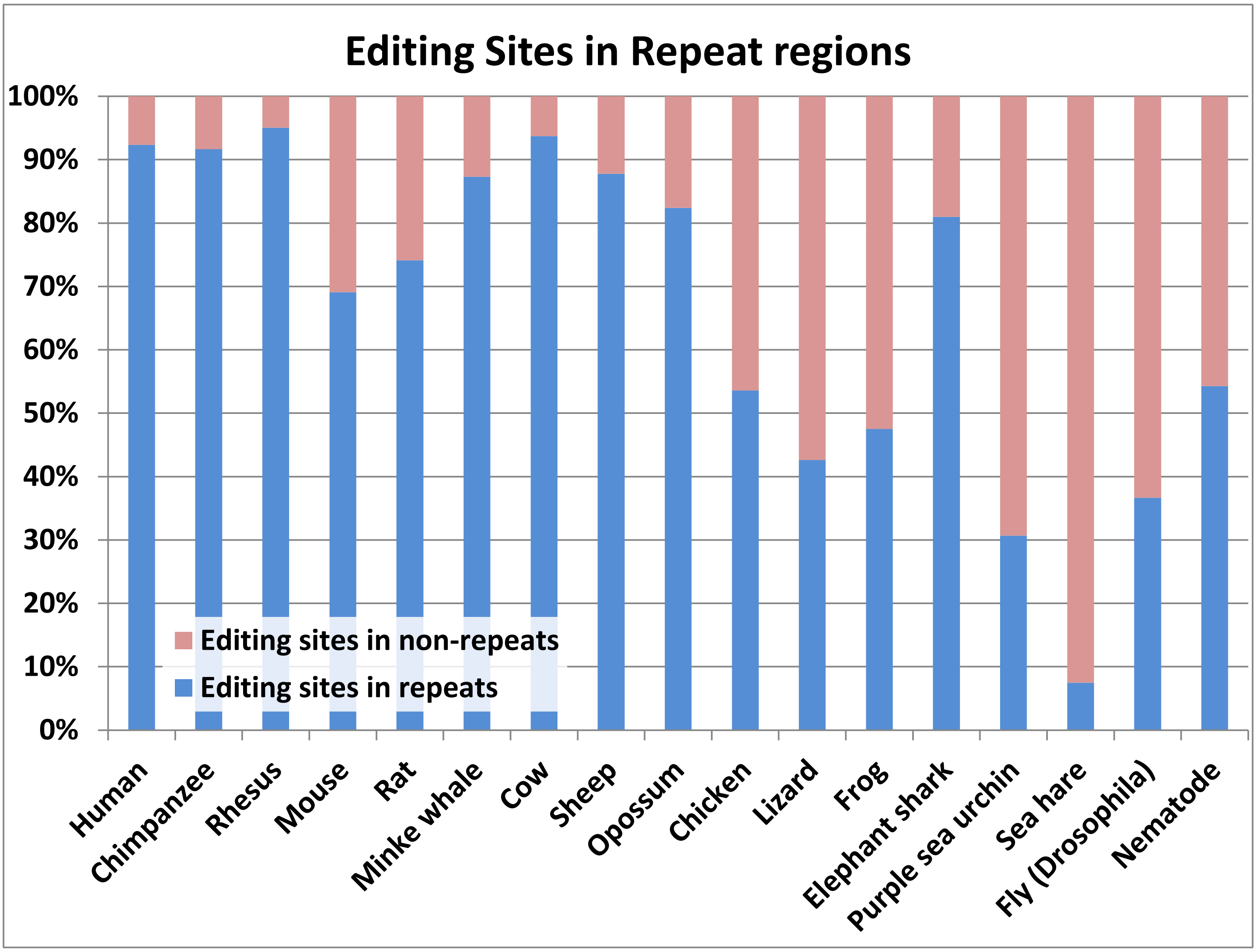

Supplementary Figure 4. **Editing sites in repeat regions.** Detected hyper-edited sites are localized mainly in repetitive elements. In most of the species, more than 70% of the sites overlapping with annotated repeats. Repeats annotations accuracy varies across species (remarkably inferior for the less researched animals), and may explain the low percentage of overlapping in some of them.

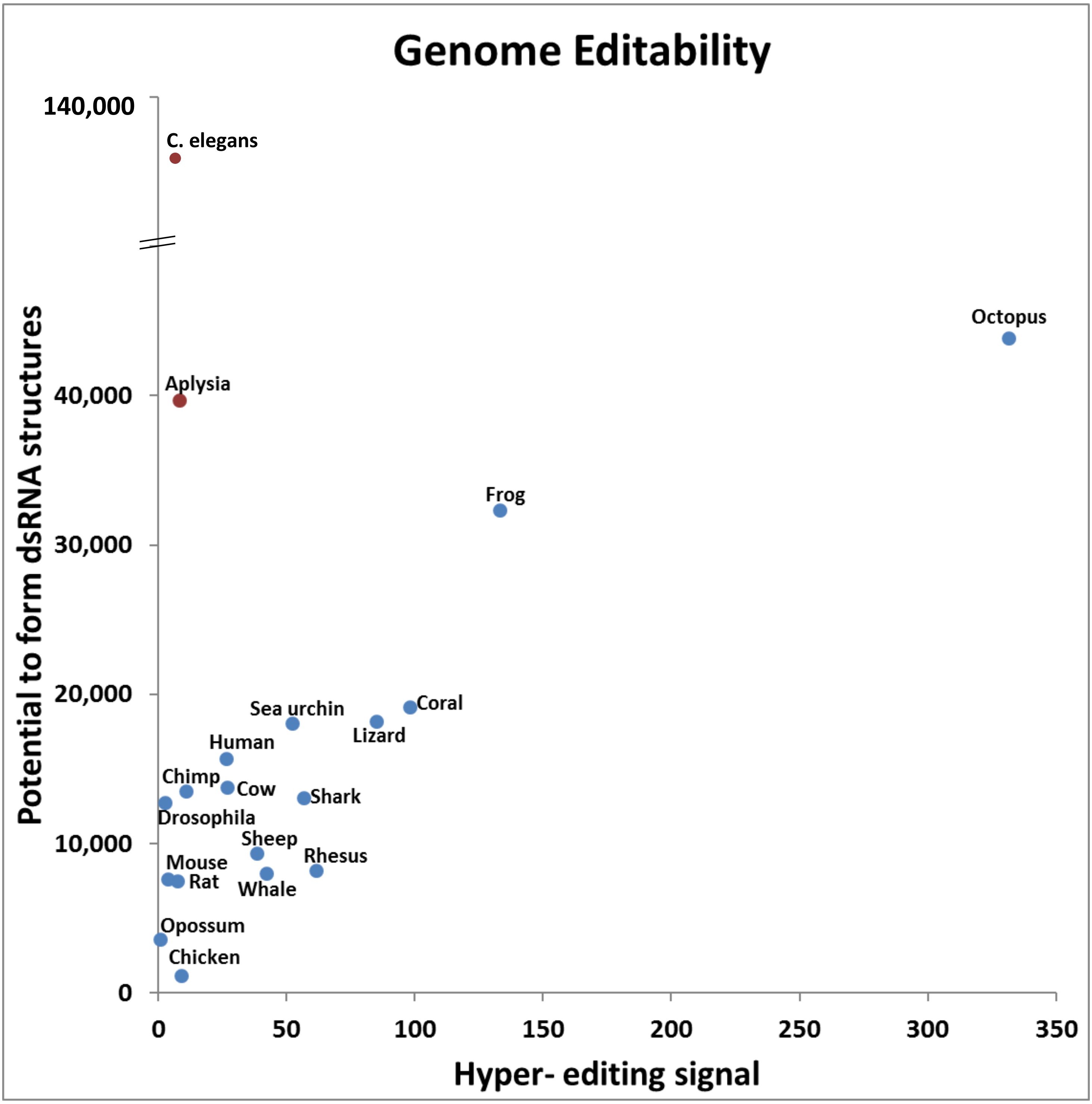

Supplementary Figure 5. The normalized hyper-editing signal correlates with the genome potential to form dsRNAs. For each genome studied, we determined the probability of a random 50bp long genomic region to form a long, nearly perfect, dsRNA (≥95% identity along 40 bp) by pairing with a reversely oriented, neighboring genomic sequence (up to 2 kb apart) (see Methods). Note that a very high potential to form dsRNA is observed for both aplysia and c. elegans, markedly higher than

expected. This exception is not explained by the editability measure and remains unclear.

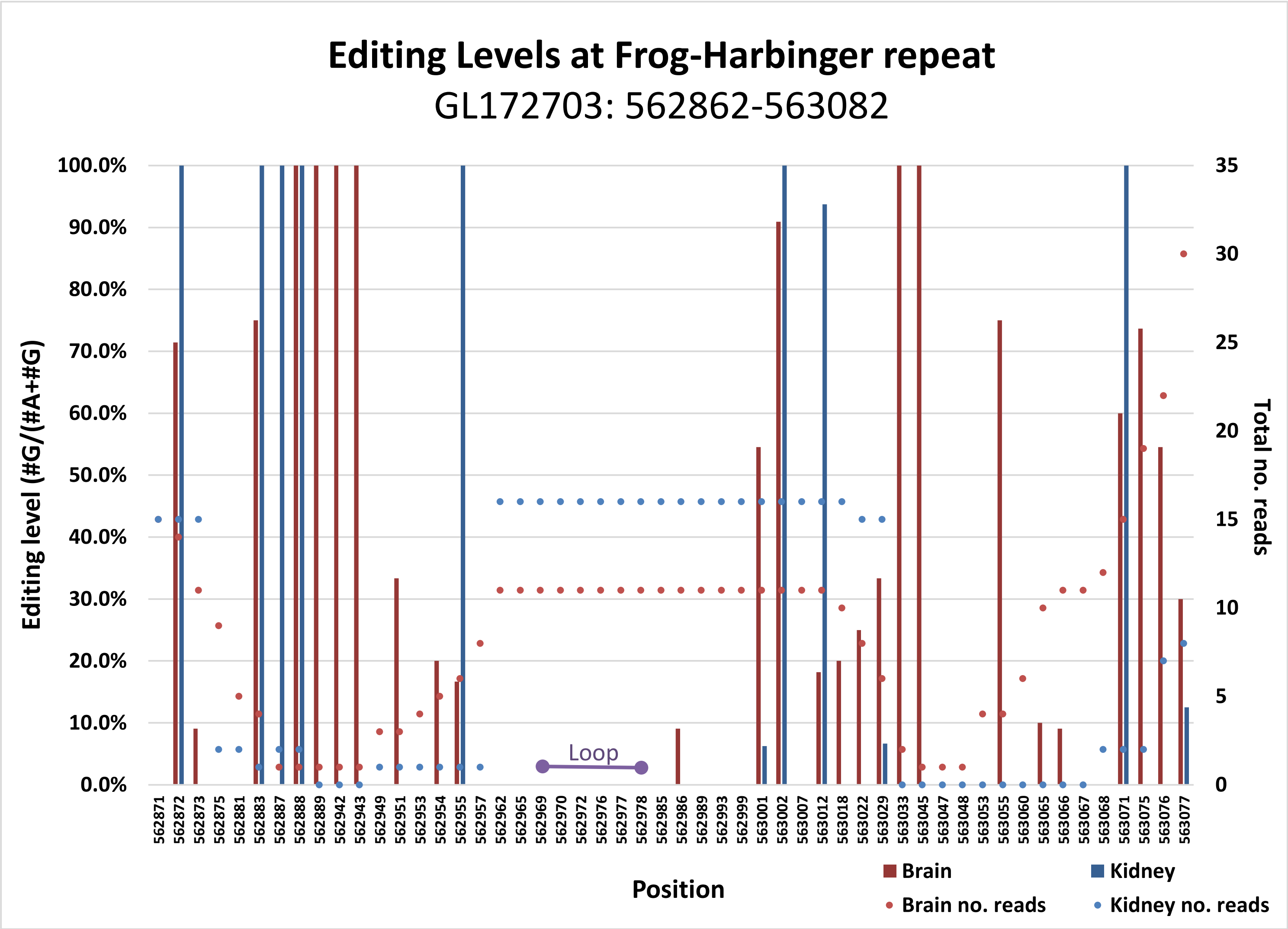

Supplementary Figure 6. Editing levels at a representative *Harbinger* repeat element from frog. *Harbinger* is the most edited repeat family in *Xenopus tropicalis*, belonging to the DNA repeat class. The *Harbinger* repeats are palindromic, likely forming tight dsRNA structures (see Figure 5). Here we show reads coverage and editing levels of the adenosines in an *Harbinger* repeat (221 bp long; located at GL172703: 562862-563082) that was found to be highly hyper-edited. The coverage and editing levels were computed using the originally mapped reads (non-hyper-edited reads) in both brain and kidney RNA-seq samples of *Xenopus tropicalis*. Results show that editing is very prevalent in the representative *Harbinger* region, even for the non-hyper-edited reads. Note that, as expected, the predicted loop region (marked in the graph, see also Figure 5) was not found to be edited.

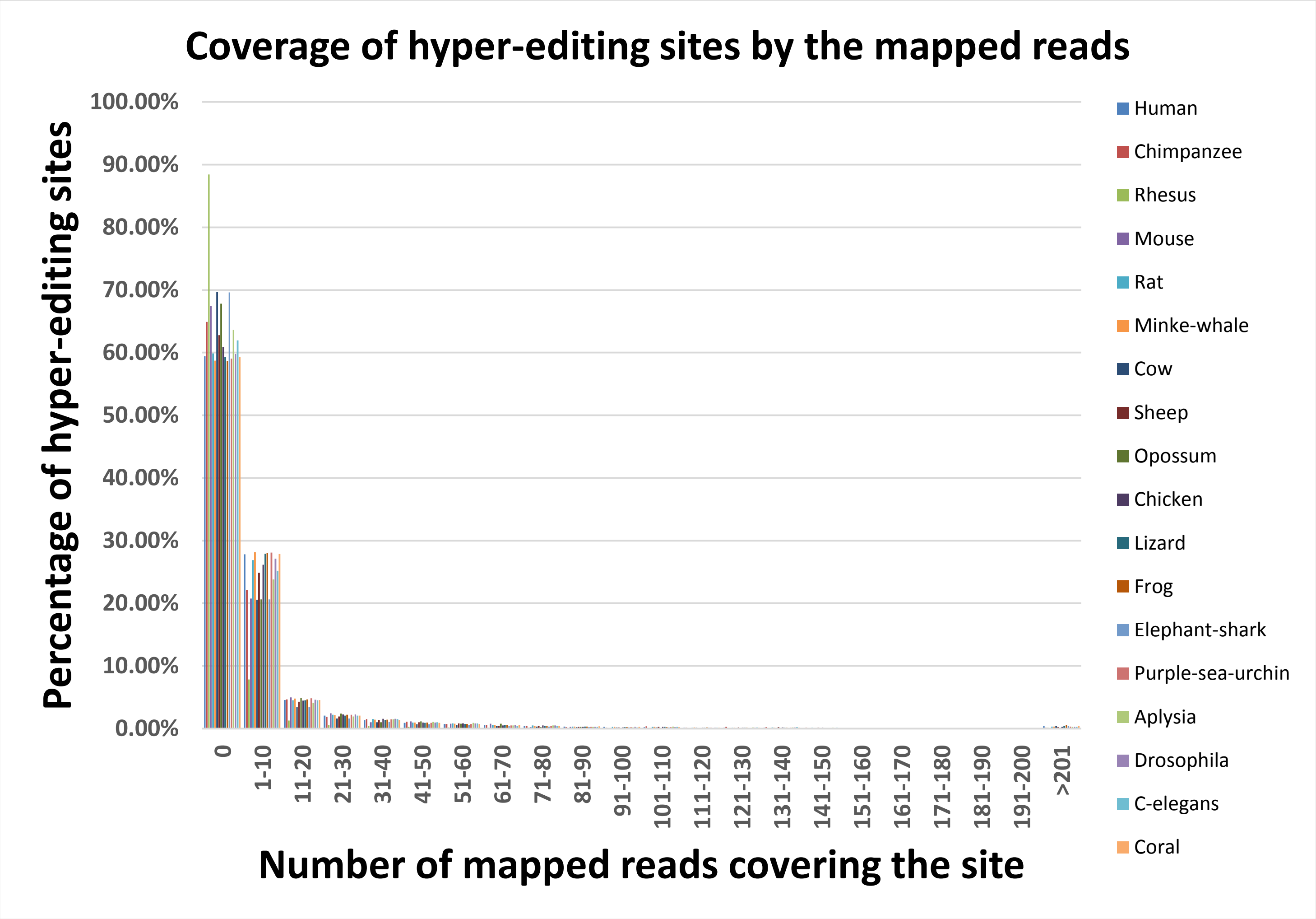

Supplementary Figure 7. Coverage of the hyper-editing sites by non-hyper-edited reads is very low. Most of the detected hyper-edited sites are not covered (and the others are lowly covered) by reads that were originally mapped to the genome. Thus, the hyper-edited regions are generally lowly expressed, and (for most regions) when expressed the resulting transcripts are nearly always hyper-edited.

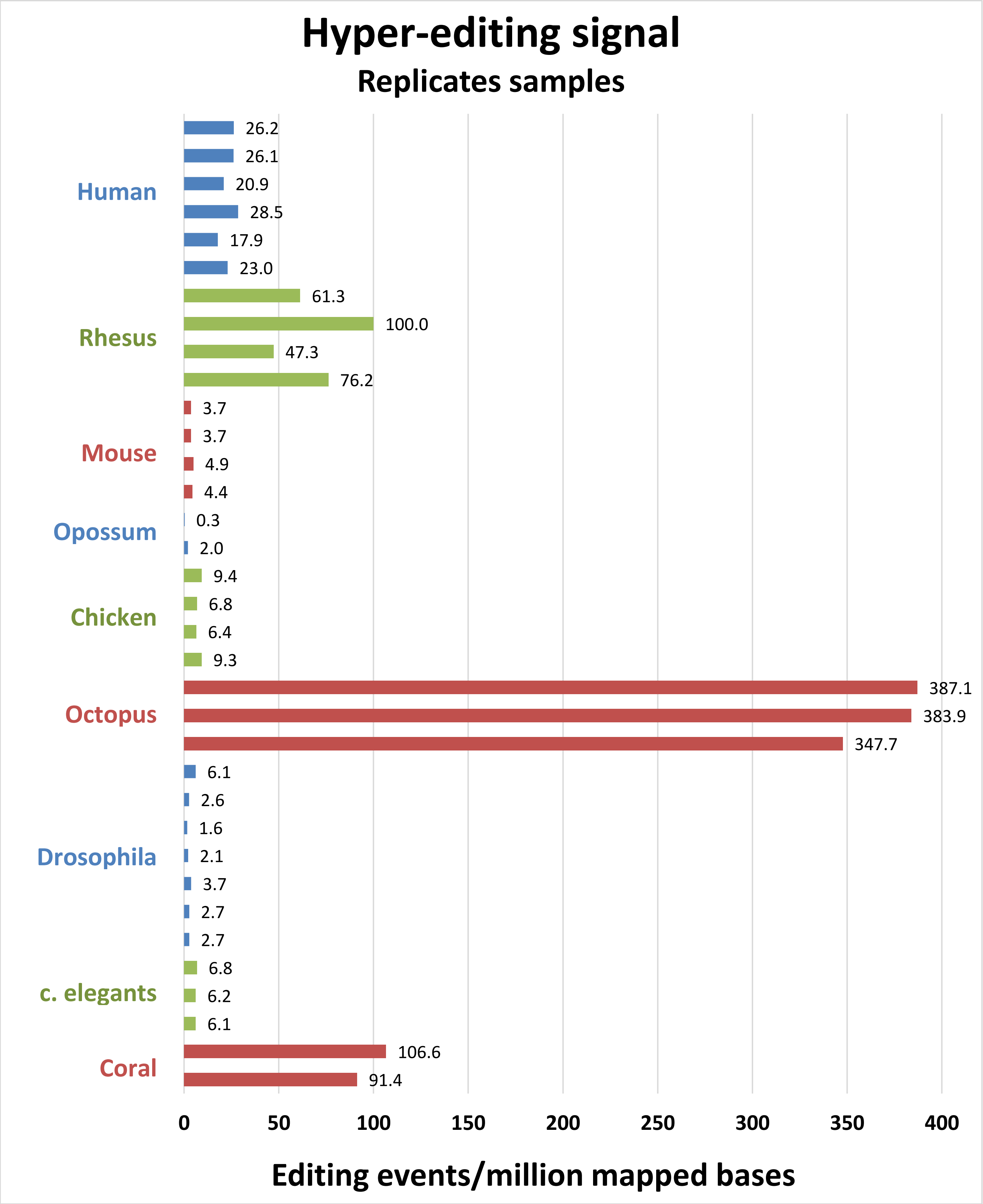

Supplementary Figure 8. Inter-species variation in hyper editing is much larger than the intra-species sample-to-sample variation. Hyper-editing normalized signals were measured for 35 RNA-seq samples of 9 organisms for which we had at least 2 biological replicates per organism. Results show that hyper-editing signals are

consistent across replicates of same species (in some cases, slightly difference tissues, see Supplementary Table 1), and are small compared with the dynamic range across metazoan species. RNA-seq reads for this analysis, were obtained from the NCBI short read archive (SRA), with the following accessions: Human- SRR309139-40, SRR2557124-7 and ERR030890; Rhesus- SRR594455, SRR649368, SRR630492 and NHPRTR (Pipes et al., 2013); Mouse- ERR033015-6 and SRR579545-6; Opossum- SRR306743-4; Chicken- SRR594500, SRR649385, ERR348563 and ERR348584; Octopus- SRR2047120, SRR2047118 and SRR2048495; Drosophila- SRR485860-3, SRR384905, SRR384939, SRR384919, SRR384959 and SRR384924-5; c. elegants- SRR1174009-11; Coral- SRR1853176 and SRR1853192.

Supplementary Table 2. Accession numbers of ADAR protein sequences used for analysis of variation in the position interacting with downstream G in PDB structure 5HP2.

Motif group	Organism	ADAR1	ADAR2	ADAR3	ADAR-like	ADAR-like (2)
**Strong downstream G preference**	Mouse	NP_062629.3	NP_001020008.1	NP_443209.2		
	Chimp	XP_513841.3	NP_001122104.2	XP_003312479.1		
	Rat	NP_112268.1	NP_037026	NP_579836.1		
	E.Shark	NA	XP_007888827	XP_007899400.1		
	Lizard		XP_008107413.1	XP_008110527.1		
	M.Whale	XP_007178544	XP_007172792	XP_007167528		
	Sheep	XP_014945675.1	XP_011959891			
	Chicken	XP_004948316.1	NP_001104544.1	XP_418563.4		
	Cow	XP_015317998.1	XP_005202097.1	NP_001179517		
	Human	NP_001102.2	NP_001103.1	NP_061172.1		
	Rhesus	XP_014966272	XP_002803150.1	XP_015001839		
**Weak downstream G preference**	Urchin	XP_001183590	XP_781832			
	Frog	XP_002943575.2	NP_001096190.1	XP_002935222.2		
	Octopus				XP_014770575.1	XP_014773875.1
	Opossum	XP_007482060		XP_007504768		
	Drosophila	NA	NP_001245474.1			
	Aplysia				XP_012939213.1	XP_005107468.1
	Coral*					
	C.elegans		NP_498594.1	NP_492154~		

*Coral's ADARs were retrieved from Porath et al 2017
